# Histopathology reveals concealed aortic valve inflammation

**DOI:** 10.1186/s13019-024-02587-0

**Published:** 2024-02-02

**Authors:** Mona Laaksonen, Ivana Kholova, Timo Paavonen, Ari Mennander

**Affiliations:** 1 Faculty of Medicine and Health Technology, Tampere University Heart Hospital and Tampere University, SDSKIR, Elämänaukio 1, P.O. Box 2000, 33521 Tampere, Finland; 2 Fimlab Laboratories, Department of Pathology, Faculty of Medicine and Health Technology, Tampere University Hospital and Tampere University, Tampere, Finland

**Keywords:** Aortic valve inflammation, Endocarditis, Aortic valve surgery

## Abstract

**Background:**

The extent of aortic valve inflammation in patients undergoing aortic valve replacement (AVR) is unsettled. The significance of aortic valve histopathology in patients undergoing AVR is undetermined.

**Methods:**

A total of 145 resected aortic valves of consecutive patients undergoing surgery for a local aortic valve disease with or without ascending aorta were investigated for histopathology. The extent of inflammation and degeneration were investigated. Unadjusted survival was evaluated by Kaplan–Meier analysis. Median follow-up was 2.7 years (interquartile range 1.5–3.9).

**Results:**

Mean patient age was 69 (SD 11) years. Though endocarditis was apparent in only six patients preoperatively, severe aortic valve inflammation was diagnosed histologically in 32 patients of whom 12 patients had acute, subacute or chronic endocarditis. Despite complete aortic valve resection, survival was decreased in patients with severe aortic valve inflammation as opposed to those without (log rank, *P* = 0.044), even after exclusion of patients with endocarditis, emergency and aortic surgery.

**Conclusions:**

Aortic valve tissue analysis reveals severe inflammation that may require postoperative treatment. The association of severe but local aortic valve inflammation with patient outcome after aortic valve surgery merits further investigation.

## Introduction

Planning for surgery of the aortic valve includes preoperative imaging techniques such as echocardiography, computed tomography (CT) and laboratory analysis [1–3]. Occasionally, accurate diagnosis of aortic valve disease underlying aortic valve stenosis or regurgitation remains challenging [4]. E.g. local aortic valve inflammation or infection may be associated with either aortic valve stenosis or regurgitation, and both surgery and postoperative care may be influenced [5]. While preoperative diagnosis of endocarditis is based on clinical criteria, definite diagnosis of endocarditis may also be confirmed solely upon histological examination of the diseased aortic valve [6, 7]. Yet it is unclear whether diagnosis of local inflammation of the resected aortic valve has clinical implications.

Traditional surgery enables complete aortic valve tissue resection and analysis. Purposeful patient care after aortic valve surgery includes follow-up of the patient. We investigated tissue analysis of the resected aortic valve and the early postoperative outcome of patients undergoing either hemisternotomy or full sternotomy for surgery of the aortic valve. The aim of the study was to identify whether aortic valve inflammation is present in patients undergoing aortic valve replacement for aortic valve disease.

## Methods

### Study protocol and surgery

After institutional review board approval (Ethical Committee of the Tampere University Hospital, Tampere, Finland, R15013), the need for informed consent was waived and the study conforms to the ethical guidelines of the Declaration of Helsinki. The aortic valve resection of 145 consecutive patients undergoing surgery for aortic valve stenosis, regurgitation or preoperative local endocarditis operated by a single surgeon during September 2015 to March 2021 in Tampere was obtained and processed for histology. Local aortic valve disease and ascending aortic dilatation were preoperatively confirmed and evaluated with CT and echocardiography. Preoperative and local aortic valve endocarditis was diagnosed according to clinical criteria [6]. Definite diagnosis of endocarditis included histopathological evaluation [6]. According to our Institutional policy, aortic dilatation included an aortic diameter more than 5.0–5.5 cm wide or aortic growth more than 1 cm in a year. This definition was adjusted to the presence of Marfan syndrome, sex, patient size and symptoms according to The Yale Center criteria [8].

The decision on the extension of resection and surgical technique was at the discretion of the operating surgeon. The aortic valve was completely resected and replaced using a bioprosthesis or a mechanical prosthesis. Hemisternotomy was considered whenever surgery encompassed AVR and aortic root replacement. Full sternotomy was performed in emergent cases and whenever extensive resection of the ascending aorta together with resection of the aortic valve, or concomitant surgery, such as coronary artery bypass grafting, mitral or tricuspid valve replacement or repair were needed. When aortic dilatation, including the sinotubular junction, was estimated as the reason for aortic regurgitation, suitable graft in a supracoronary fashion was tailored. Whenever dilatation included the aorta root, a radical resection of the dilated ascending aorta, together with the root and the aortic valve, was performed. The graft size was estimated by the surgeon. The whole aortic valve was procured and processed for tissue analysis.

### Histology and immunohistochemistry

**Fig. 1:**
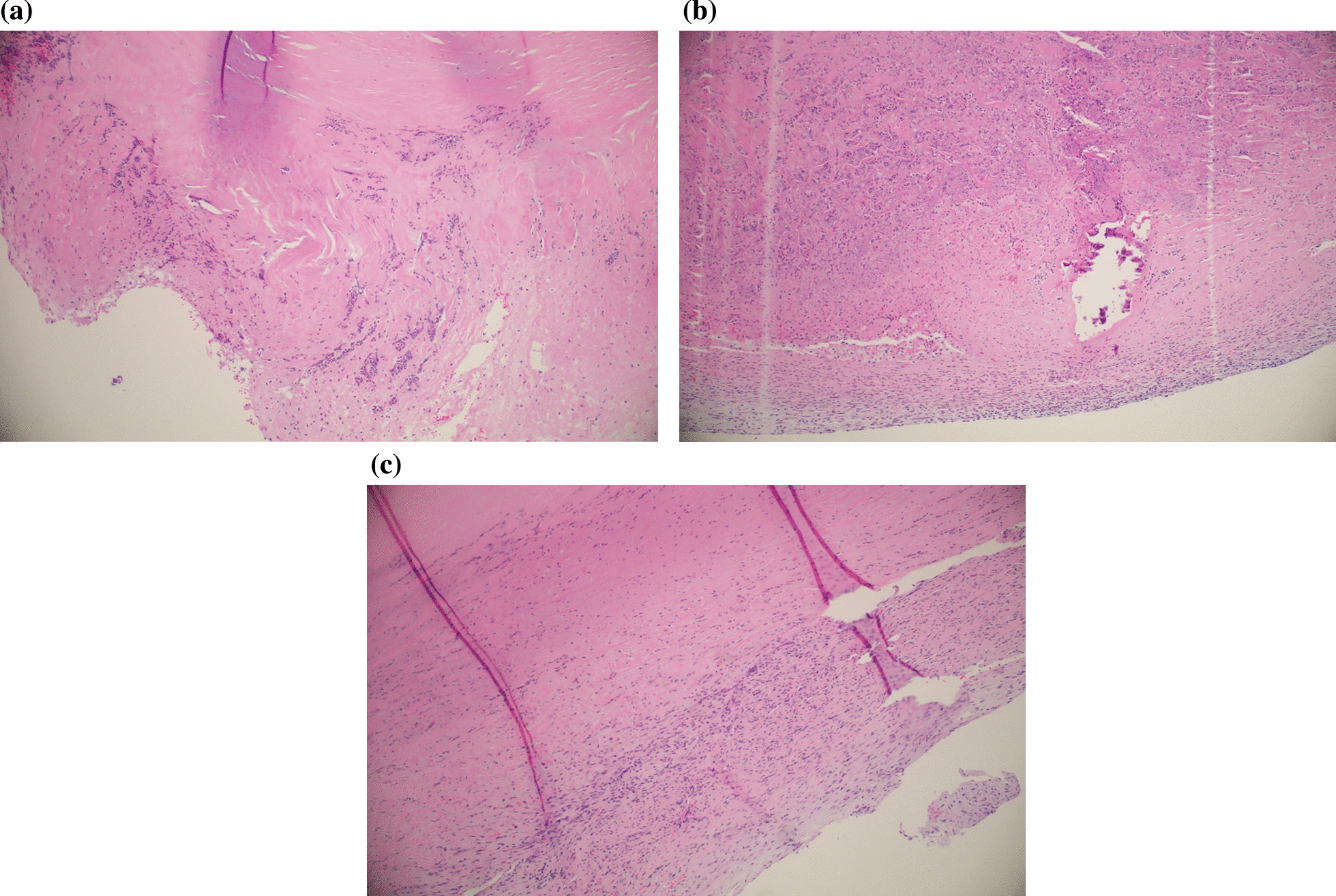
Representative aortic valve histology showing inflammation and degeneration (Hematoxylin–eosin, 100 × magnification). Focuses of neutrophils dominate the inflammatory pattern during acute infective endocarditis (**A**). Moderate mixed infiltration of lymphocytes and neutrophils with acellular debris and bacteria during subacute endocarditis (**B**). Chronic endocarditis characterized by chronic infiltration of lymphocytes (**C**)

Two to six pieces of resected aortic valve were embedded in paraffin, cut to 4 μm thick segments and stained with Hematoxylin and Eosin, Verhoeff-van Gieson, Acian Blue and Periodic Acid-Schiff. Aortic valve cusps corresponding to all different staining were evaluated systematically for all resected samples procured during surgery (Fig. 1).

### Quantification of histopathology

The configuration of the aortic valve was recorded encompassing the number of cusps during surgery. Medial degeneration and presence of calcium deposits were evaluated semi-quantitatively. We used the following semi-quantitative criteria for the evaluation of medial degeneration: none = no signs of degeneration; mild = occasional disruption of elastic fibers, sparse presence of either myxoid matrix or fibrosis; moderate = larger changes described above; severe = diffuse and severe degeneration; and for aortic valve calcium deposits: none = no calcium, mild = single calcium deposit, moderate = patches of calcium deposits, severe = diffuse calcium deposits. The presence of moderate to severe inflammation was recorded. Definite local endocarditis was defined as acute infection with vegetations, subacute, and chronic [9–12] Two experienced pathologists (IK and TP) evaluated the samples.

### Follow-up protocol

Documentation of mortality and morbidity was available for all the patients. For the included study patients, follow-up consisted of physical examination and echocardiography at three months after surgery, and on-demand thereafter including computed tomography. Morbidity after surgery included cerebral stroke, dialysis, and mediastinitis.

### Statistical analysis

Continuous variables were expressed as means with standard deviations and were compared using the Mann–Whitney test. Median and interquartile range are provided for follow-up times. Categorical variables were presented as numbers and percentages and were compared using χ^2^ or Fisher’s exact tests. The outcome of patients with histologically confirmed aortic valve inflammation were compared with those without inflammation. Multivariable Cox proportional hazards regression model adjusted for endocarditis, emergency, aortic surgery, and age was performed to assess associations between aortic valve inflammation without endocarditis and mortality. Unadjusted survival was evaluated by Kaplan–Meier analysis with log-rank test for all patients, and when endocarditis, emergency and aortic surgery cases were excluded. All analyses were conducted using the IBM SPSS Statistics version 28.0 (IBM Corporation, Armonk, NY, USA).

## Results

### Patient characteristics

Patient characteristics are shown on Table 1. There were 145 patients of which 38 (26%) patients needed emergent or salvage surgery. Preoperative endocarditis was diagnosed clinically in six patients (4%) before surgery. There were six aortic dissections and 23 patients had aortic dilatation. The mean age for the patients was 69 years. Almost a double of the patients were men, body mass index was 28 and EuroScore II was 6.4. Hypertension was presence in a third of the patients. The mean aortic valve annulus diameter was 23 mm. There were 69 patients with aortic valve stenosis, 24 aortic valve regurgitation and 52 combined aortic valve regurgitation with stenosis. Median follow-up was 2.7 years (interquartile range 1.5–3.9).

**Table 1:** Patient characteristics and preoperative data

	All patients
Number of patients	145
Age, years	69 ± 11
Male, n	92 (64%)
Body mass index, kg/m^2^	37 ± 41
Aortic valve stenosis, n	69 (48%)
Aortic valve regurgitation, n	24 (17%)
Aortic valve stenosis and regurgitation, n	52 (36%)
Aortic valve size, mm	23 ± 2
Preoperative endocarditis, n	6 (4%)
Current smoker, n	80 (55%)
Ex-smoker, n	17 (12%)
Non-smoker, n	48 (33%)
Hypertension, n	50 (35%)
Diabetes, n	18 (13%)
Dyslipidemia, n	45 (31%)
Known coronary artery disease, n	1 (1%)
Family history of heart disease, n	23 (16%)
Ejection fraction, %	55 ± 13
Euroscore II, %	5.9 ± 12.4
Glomerular filtration rate, %	62 ± 32
Elective, n	107 (74%)
Emergency, n	25 (17%)
Salvage, n	13 (9%)
Redo surgery, n	1 (1%)
Aortic dissection, n	6 (4%)
Aortic dilatation, n	23 (16%)

### Operative technique

The operative technique is shown on Table 2. Ascending aorta replacement was performed in 29 (20%) patients together with AVR, while 116 (80%) patients underwent AVR only. A third of the patients were operated through a hemisternotomy, while full sternotomy was performed whenever salvage or emergent surgery was required. A biological valve was implanted with or without a conduit prosthesis in the majority of the patients (130 out of 145, 90%) as opposed to only 15 out of 145 (10%) patients with a mechanical valve prosthesis with or without aortic replacement. Patients that had additional surgery besides aortic valve replacement included 27 concomitant coronary artery bypass grafting, eight mitral valve replacements, four tricuspid valve plastia, one atrial septal closure and two patients had concomitant atrial septal resection caused by hypertrophic obstructive cardiomyopathy.

**Table 2:** Operative details according to surgical evaluation of extension of diseased aorta

	All patients
All operations, n	145
Aortic valve replacement, n	116 (80%)
Aortic valve replacement + aortic prosthesis, n	29 (20%)
Aortic valve replacement, n	145
Mechanical, n	10 (7%)
Biological, n	110 (76%)
Rapid deployment Intuity®, n	41 (28%)
Mechanical conduit, n	5 (3%)
Biological conduit, n	20 (14%)
Incision, n	145
Hemisternotomy, n	49 (34%)
Sternotomy, n	94 (64%)
Conversion, n	2 (2%)
Cardiopulmonary bypass time, min	171 ± 88
Aortic cross-clamp time, min	130 ± 58
Cardioplegia	
Antegrade, min	7 ± 7
Retrograde, min	20 ± 18

### Perioperative findings, histology and immunohistochemistry

As shown on Table 3, the majority of the patients had tricuspid aortic valves. Aortic valve calcification of the aortic valve was present in 125 patients (86%), and degeneration was moderate to severe in 81 patients (56%). Altogether, severe aortic valve inflammation was found in 32 patients (22%), of which 12 (9%) had definite endocarditis including seven active aortic valve endocarditis with acute infection, two subacute and three chronic aortic valve endocarditis.

**Table 3:** Histology and quantitative immunohistochemistry

	Preoperative endocarditis	Concealed endocarditis	Inflammatory aortic valve	Non-inflammatory aortic valve
Aortic valve cups, n	6	6	20	113
Tricuspid, n	3	3	10	80
Bicuspid, n	3	3	10	31
Unicuspid, n		–	–	2
Calcification, n	6	6	20	113
None	1	1	2	16
Mild, n	2	2	0	11
Moderate, n	–	–	1	19
Severe, n	3	3	17	67
Degeneration, n	6	6	20	113
None	–	–	7	24
Mild, n	1	1	2	22
Moderate, n	1	1	3	57
Severe, n	4	4	8	3
Inflammation, n	6	6	20	–
Definite endocarditis, n	6	6	–	–
Acute/vegetative/infective, n	6	1	–	–
Subacute, n	–	2	–	–
Chronic, n	–	3	–	–

### Morbidity

There were five patients with postoperative stroke, four dialysis and two mediastinitis. Early 30-day mortality occurred in eight patients (Table 4). There were no reoperations during follow-up.

**Table 4:** Postoperative outcome

	All patients N = 145	Preoperative endocarditis	Concealed endocarditis	Inflammatory aortic valve	Non-inflammatory aortic valve
Stroke	5 (3%)	–	–	–	5
Dialysis	4 (3%)	–	1	–	3
Mediastinitis	2 (2%)	–	–	–	2
Sternal dehiscence	2 (2%)	–	–	–	2
30-day mortality	8 (6%)	–	2	1	5

### Survival

**Fig. 2:**
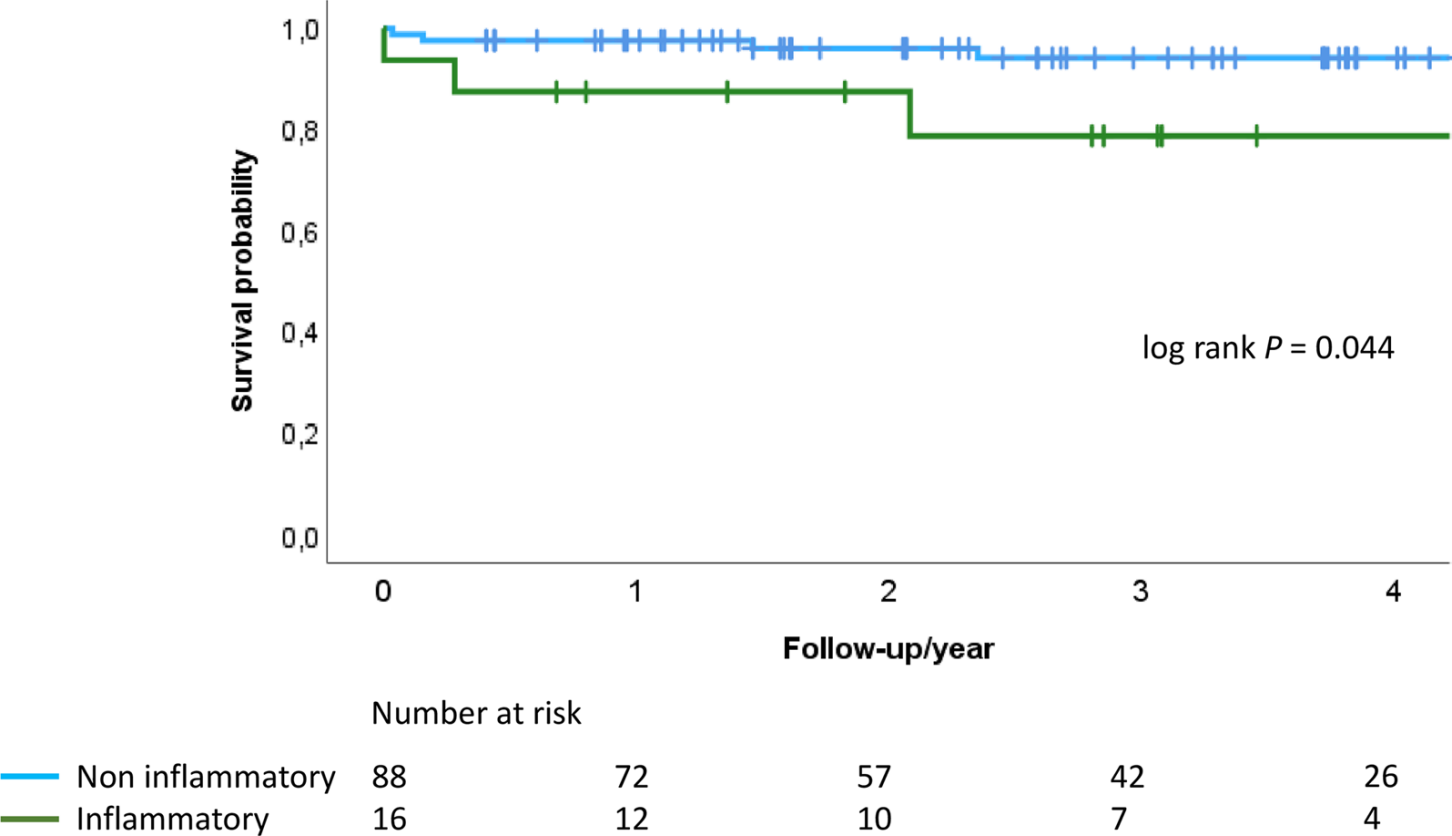
Survival probability of patients after aortic valve surgery without (*blue line*) and with aortic valve inflammation (*green line*). Time-varying outcome according to Kaplan–Meier estimation. Log rank *P* = 0.044. Patients with endocarditis, emergency and aorta surgery are not included

According to Kaplan–Meier analysis, survival differed between all patients with aortic valve inflammation vs not (log rank *P* = 0.046). Altogether, 15 patients died during follow-up, of which six patients had aortic valve inflammation. All-cause survival was lower in patients with emergency (adjusted hazard ratio (aHR), 8.05; 95% confidence interval [CI], 2.04–31.86, *P* = 0.003) and aortic surgery (aHR, 3.24; 95%CI, 1.06–9.92, *P* = 0.039), but not with endocarditis per se (aHR, 2.21; 95%CI, 0.43–11.40, *P* = 0.342); survival was marginally lower with age (aHR, 1.05; 95%CI, 1.00–1.11, *P* = 0.056) and aortic valve inflammation without endocarditis (aHR, 3.04; 95%CI, 0.83–11.18, *P* = 0.094). After exclusion of endocarditis, emergency and aortic surgery patients, survival still differed between patients with only aortic valve inflammation vs not (Fig. 2, log rank *P* = 0.044).

## Discussion

This contemporary study shows that aortic valve inflammation is present in almost a quarter of patients undergoing AVR with or without replacement of the ascending aorta. Definite aortic valve endocarditis was observed in 12 patients including three chronic endocarditis, whereas only six of these were preoperatively diagnosed. Tissue analysis of the aortic valve not only confirms endocarditis, but also reveals concealed inflammation that may explain the pathophysiology of the aortic valve dysfunction.

Patients undergoing AVR have a multifactorial presentation of clinical symptoms [13, 14]. Despite different patient characteristics, current surgical treatment includes resection of the diseased aortic valve including valve stenosis, calcified and inflammatory tissue. Aortic valve pathology may also reflect the overall clinical state of the patient [15, 16]. The adjacent dilated or diseased aorta may also need concomitant surgery [17].

Active, subacute or chronic endocarditis may account for ongoing atherosclerosis, progression of tissue calcification or even concealed inflammation necessitating antibiotics after surgery [7]. As risk factors for aortic valve disease, such as hypertension, male sex, family history of aortic aneurysm, diabetes, smoking and coronary artery disease were present in all patients [13], it is preoperatively difficult to estimate the extent of aortic valve inflammation without clinical signs of endocarditis such as fever, sepsis, malaise or multiorgan failure [18]. Ideally, preoperative diagnosis of active endocarditis includes the identification of the pathogen by blood culture [19], while aortic valve histopathology confirms the extent of tissue destruction and inflammation [7]. However, diagnosis of culture-negative endocarditis may even be impossible without histology [6, 7, 20]. Valve culture yields an only 13% sensitivity as opposed to a 63% sensitivity and 100% specificity for histological analysis in detecting endocarditis [21].

Inflammation itself is associated with aortic valve degeneration including atherosclerosis and calcification, adding to functional heterogeneity such as aortic valve stenosis and regurgitation [15]. The presence of inflammation is crucial during amyloidosis that may lead to e.g. aortic stenosis [22]. Clinically, it remains to be shown whether inflammation per se without endocarditis would necessitate additional treatment after resection of the diseased valve [23]. Even after aortic valve resection, we observed decreased survival in patients undergoing AVR with inflammation as compared to those without. Clinically, patient surveillance is important after surgery [23]. Indeed, postoperative antibiotic treatment was considered to our AVR patients after degenerative and inflammatory features of the aortic valve were confirmed histologically [4, 7, 24].

Currently, the feasibility of implanting a valve inside the degenerative native aortic valve without surgical resection (transfemoral or transapical aortic valve replacement) is an attractive choice for many comorbid patients, but long-term follow-up of these patients is still pending [25]. Indeed, avoiding resection and debridement of the diseased and inflammatory tissue may lead to increased paravalvular leakage, occasionally obstruction of the coronary ostia or emboli of aortic valve debris during aortic valve implantation [26, 27]. The presence of degenerative or inflammatory histological features justifies complete resection of the frail aortic valve at least when long-term outcome is expected [4]. Impeccable function of the implanted aortic valve prosthesis is anticipated, while the diseased aortic valve is completely resected [5, 23]. We resected the diseased aortic valve in all the patients despite using either hemisternotomy or full sternotomy.

## Conclusions

Aortic valve histology confirms tissue degeneration and inflammation that may reveal definite diagnosis of endocarditis. Complete resection and histology of the diseased aortic valve adds to decision-making for plausible postoperative medication.

## Limitations

This study represents a real-life single-center contemporary cohort. The limitations of this study include the small number of patients with a relatively short follow-up, and aortic valve histology is obviously only available in patients that underwent surgery. Based on the design of the study, we excluded patients undergoing complex surgery for extensive endocarditis encompassing other cardiac valves, abscess formation and presence of sepsis.

## Abbreviations

AVR: Aortic valve replacement
CT: Computed tomography
IQR: Interquartile range
SD: Standard deviation
